# Superoxide Dismutases (SODs) and SOD Mimetics

**DOI:** 10.3390/antiox7110156

**Published:** 2018-11-02

**Authors:** Gloria E. O. Borgstahl, Rebecca E. Oberley-Deegan

**Affiliations:** 1 Department of Biochemistry and Molecular Biology, University of Nebraska Medical Center, Omaha, NE 68198, USA; 2 Eppley Institute for Cancer and Allied Diseases, 986805 Nebraska Medical Center, Omaha, NE 68198-6805, USA

Superoxide dismutase (SOD) is the only known enzyme to directly scavenge a free radical. Specifically, SOD dismutes superoxide to oxygen and hydrogen peroxide with high specificity and efficiency. Since its discovery in 1969 by Dr. Irwin Fridovich, SOD has been shown to be present in every living organism on Earth and, thus, essential for life. Mammalian cells have three forms of SOD: Cu/ZnSOD, mainly found in the cytosol; MnSOD, located in the mitochondria and ECSOD, detected in the extracellular space. SOD enzymes regulate the levels of superoxide and hydrogen peroxide produced by cells, which in turn regulates cell signaling. Depending on the location of the SOD enzyme, these proteins protect extra- and intracellular structures from oxidation and damage. 

In the past 50 years, it has become increasingly evident that SOD plays a major role in maintaining cellular health, and a variety of diseases occur if SOD becomes dysregulated. SOD enzymes are highly regulated through modifications at the transcriptional, translational, and post-translational level. Genetic mutations in SOD proteins have also been associated with a variety of diseases. Recent studies also indicate that SOD activity is altered in disease states even while changes in SOD protein levels are not evident. 

This special issue highlights current research concerning SOD and the role that SOD mimetics play in boosting superoxide scavenging in both normal and diseased states. In work directed at normal cellular processes, Dr. Case reviews the origin of superoxide dismutases and how evolution has shaped superoxide-mediated redox signaling [1]. In another review by Azadmanesh et al. [2], the active site of MnSOD is characterized via crystallography and the catalytic mechanism of action of MnSOD is described in detail. Yarana et al. [3] review the effects of extracellular vesicles communicating damage to other cell types. Some recent findings indicate that SOD proteins can be transferred between cells through these extracellular vesicles, which could provide another way in which SOD proteins are regulated. In a primary research article, Kalen et al. [4] demonstrate the coordination between MnSOD and Cyclin B1 during cell cycle response to oxidative stress in normal fibroblasts. Specifically, they demonstrate cross-talk between the mitochondria and the nucleus to cause mitochondrial-checkpoint cell arrest when cells encounter oxidative stress. Finally, in another primary research study, Leong et al. [5] demonstrate that the addition on a SOD-like drug protects normal cells from oxidant-induced injury.

This issue also highlights the role of SOD in a variety of diseases, such as cancer, diabetes, pulmonary hypertension, bronchopulmonary dysplasia, radiation-induced fibrosis, and arthritis. There are several articles dedicated to SOD and cancer and the use of SOD mimetics as therapeutics against cancer progression. Wilkes et al. [6] review the role of SOD-induced inhibition of tumor growth and propagation and its potential as a target in pancreatic cancer. Kim et al. [7] review the dichotomous regulation of MnSOD in cancer and describe the mechanism of regulation of MnSOD in cancer, with an emphasis on post-translational modifications. This review is focused on understanding the spatiotemporal nature of MnSOD regulation in the context of a changing tumor microenvironment, which is necessary to improve the design of oxidant- or antioxidant-based therapeutic strategies for the treatment of cancer. In primary research articles by Chatterjee et al. [8] and Heer et al. [9], these investigators use SOD mimetics to enhance anti-cancer treatment. Chatterjee et al. [8] demonstrate that SOD mimetics, in combination with radiation, inhibit prostate cancer growth while simultaneously protecting the normal prostate tissue from radiation damage. Heer et al. [9] enhance the anti-tumor effect of pharmacological ascorbate with the addition of SOD mimetics by enhancing hydrogen peroxide levels in the tumor.

Radiation-induced fibrosis is caused in large part from free radical production, which results in damage to normal tissues. SOD overexpression has been shown to mitigate radiation-induced damage. Studies conducted by Cline et al. [10] and Shrishrimal et al. [11] demonstrate that SOD mimetics protect from radiation-induced fibrosis. In a lung fibrosis model, Cline et al. [10] demonstrate that the addition of a SOD mimic after whole thorax radiation exposure protects from lung fibrosis. In a pelvic irradiation model, Shrishrimal et al. [11] show that a SOD mimic prevented acute and chronic irradiation damage in pelvic tissues and demonstrated that SOD treatment prevents myofibroblast differentiation that is induced by radiation exposure.

MnSOD plays a critical role in maintaining redox balance in the mitochondria and thereby also helps to regulate cellular metabolism. In metabolic diseases, such as diabetes, there are high amounts of systemic oxidative stress along with dysfunctional mitochondria. Coudriet et al. [12] show that a SOD mimetic protects from liver damage, improves insulin sensitivity, and reduces inflammation associated with obesity-induced type 2 diabetes. In a chemical induced model of mitochondrial oxidative stress and altered metabolism, Alam et al. [13] demonstrate that exogenous MnSOD can overcome mitochondrial changes in SIRT3^−/−^ cells.

ECSOD is highly expressed in the lung, cardiovascular system, and cartilage. Alteration in ECSOD expression is associated with disease in these tissues, and polymorphisms of ECSOD have been identified to contribute to lung and cardiovascular disease. In the primary article by Sherlock et al. [14], the authors show that a polymorphism of ECSOD, R213G, results in more ECSOD in the serum and less ECSOD bound to the vasculature. The R213G ECSOD mice have abnormal pulmonary vascular development but are better protected from lung injury. ECSOD is highly expressed in cartilage and is thought to protect this tissue from protein oxidation and breakdown. Using impact and overload injury scenarios to bovine osteochondral explants, Coleman et al. [15] demonstrate that the superoxide is produced after high impact to cartilage tissues and the addition of a SOD mimetic protected from cartilage damage.

This special issue demonstrates the diverse functions that SOD enzymes play in normal and diseased states and highlights exciting new SOD mimetics that may provide some therapeutic strategies to prevent or lessen the severity of diseases.
